# Phytochemical constitution and antioxidant activity of functional herbal drink from Indian gooseberry (*Emblica officinalis* Gaertn.) fruits containing spices and condiments

**DOI:** 10.1186/s43014-022-00127-8

**Published:** 2023-06-01

**Authors:** Saji Gomez, C. Anjali, Bintu Kuruvila, P. K. Maneesha, Meagle Joseph

**Affiliations:** Department of Post Harvest Technology, College of Agriculture, Kerala Agricultural University, Vellanikkara, Thrissur, 680656 India

**Keywords:** Indian gooseberry, Tannins, Gingerol, Piperine, Curcumin, DPPH, FRAP, ABTS

## Abstract

Functional beverages originate primarily from fruits and vegetables sources, but also include those from other plants such as tea, coffee, cocoa, soybean as well as animal products like milk and dairy-based and alcoholic drinks. They have definite medical or health benefits which include prevention or delaying the progress of diseases. Indian gooseberry is a very rich source of vitamin C and phenolics, two potent antioxidant compounds. Similarly curcumin in turmeric, piperine in black pepper and gingerol in ginger have proven antioxidant and anti-inflammatory properties. Incidence of covid-19 pandemic has raised awareness among people the importance of maintaining higher levels of immunity. A study was undertaken at Kerala Agricultural University during 2020–21 to develop an herbal functional drink from Indian gooseberry fruit juice incorporated with turmeric and black pepper powders, ginger juice extract and juice of acid lime fruits. The herbal drink formulated with these ingredients was homogenized at an operating pressure of 175 Bar with a speed of 235 SPM and was subsequently pasteurized at 100 ^0^ C for 10 minutes in glass bottles, followed by storage under refrigerated conditions at 5 ± 2 ^0^ C for 3 months. The initial ascorbic acid, total phenolics, total flavonoids, total carotenoids and total curcumin contents were 61.0 mg100g^− 1^, 184.0 mg100g^− 1^, 153.0 mg100g^− 1^, 119.98 mg100g^− 1^ and 31.0 mg100g^− 1^, respectively. Antioxidant activity of the herbal drink was determined by three assays, viz. ABTS, DDPH and FRAP. The initial IC _50_ values of the herbal drink by ABTS, DPPH and FRAP assays were 8.64, 0.212 and 0.368 μgml^− 1^, respectively. Significant decline in ascorbic acid, total flavonoids, total carotenoids and curcumin content were recorded in the product during storage in contrast to the total phenolics content which showed a significant rise over the storage period. Antioxidant activity of the herbal drink determined by all the three assays also declined significantly throughout the storage period. The results indicate that the product can be promoted as a healthy drink which has to be stored at low temperature in order to retain higher levels of antioxidant compounds and antioxidant activity.

**Graphical Abstract:**

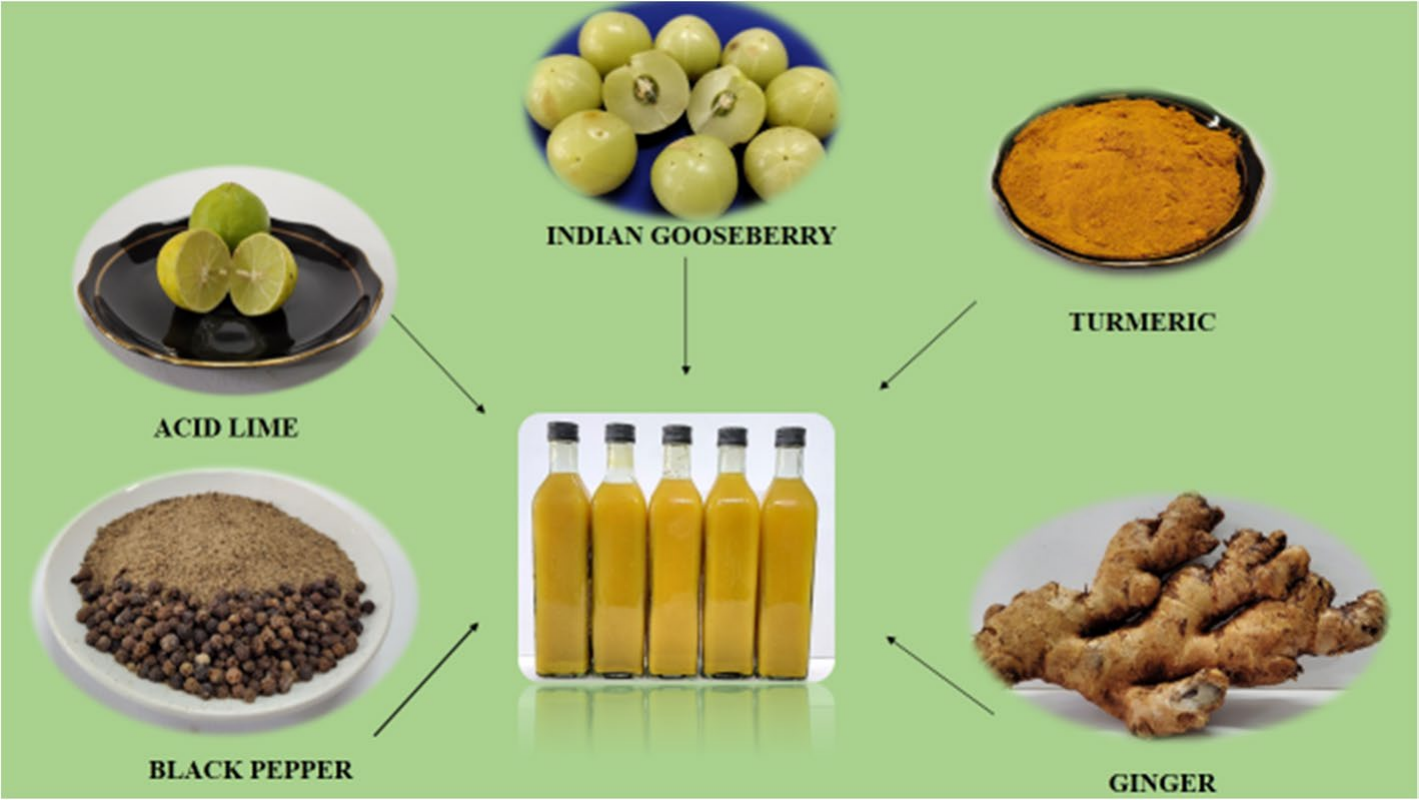

## Introduction

Functional beverages can provide definite health benefits like disease prevention or can retard progression of disease or may even check the occurrence of physiological abnormalities. Functional beverages originate primarily from fruits and vegetables sources, but also include those from other plants such as tea, coffee, cocoa, soybean as well as animal products like milk and dairy-based and alcoholic drinks (Shahidi & Weerasinghe 2003). Substances having functional properties may range from nutrients in these food items, food supplements, dietary ingredients, fruit and vegetable beverages, and also, herbal products (Keservani et al. 2010). Many of the herbs grown in various parts of the world have been found to contain specific phytochemicals which possess unique therapeutic functions. Increased awareness on nutrition and safety has made people across the globe health-conscious and this has prompted them to look for natural products free of harmful chemicals. Moreover, increase in disposable incomes and better standard of living have resulted in more options in food preferences. Besides, unprecedented occurrence of pandemics has posed severe risk in compromising immunity which make people vulnerable to such infections. Therefore, increased attention is being given to plants having specific health protective properties, particularly those having compounds that can boost immunity levels. Dietary habits have direct bearing on oxidative and inflammatory conditions of humans. Underlying etiological factors may develop into chronic diseases as a result of long-term imbalances of oxidative and inflammatory stress which could lead to damage or even failure of tissue (Joseph et al. 2015). The occurrence of various types of pandemics, especially covid-19 has reinforced the significance of maintaining higher levels of immunity to ward off pathogens responsible for such pandemics and has also made people aware the need of consumption of foods rich in antioxidants, having anti-inflammatory properties and all the more, to be ‘chemical-free’ and natural in constitution. Several methods of preservation are being adopted to extend shelf life of fruit and vegetable juices. Though thermal processing results in degradation of phytochemicals and adversely affects the sensory properties of fruit and vegetable products, it is still the most widely used and economical method of preservation of fruit juices. Thermal pasteurization is able to extend shelf-life of the juices by inactivation of microbes and undesired enzymes (Rawson et al. 2011). Indian gooseberry fruit or its juice is seldom relished by consumers in the fresh form owing to its high acidity and astringency. Hence, the study was undertaken to develop a functional herbal drink with Indian gooseberry which is a rich source of vitamin C and phenolic compounds as the base raw material, incorporated with turmeric, ginger, acid lime and black pepper, all with strong antioxidant and anti-inflammatory properties. Therefore, the main objective of the study was formulation of a unique drink from Indian gooseberry fruits with improved palatability and altered flavour and also, to test the stability of the various antioxidant compounds as well as the antioxidant activity of the drink during storage. The main purpose was to provide a herbal drink devoid of chemical preservatives but developed through ‘green technology’ which could be consumed for boosting immunity levels of consumers.

The total antioxidant capacity of food items is influenced by technological process, packing, matrices and the contribution by other antioxidant metabolites. Also, the behavior of antioxidant compounds and total antioxidant activity of functional beverages is highly variable (Castro-Lopez et al. 2016). In general, herbal beverages are prepared from natural ingredients present in fruits, roots, leaves, roots and inflorescence (Chandrasekara & Shahidi 2018). They are rich sources of natural bioactive compounds like phenolic acids, carotenoids, flavonoids, polyacetylenes, coumarins, alkaloids, saponins and terpenoids. According to Chandrasekara and Shahidi (2018), Health Canada categorizes herbal beverages under natural health products (NHPs). According to Health Canada, moderate consumption (2–3 cups/ day) of herbal beverages of lemon balm, citrus peel, rose hip and ginger are recommended during pregnancy and breastfeeding (Public Health Agency of Canada 2016). Phenolic compounds and vitamin C are vital antioxidants, contributing significantly to human health. Indian gooseberry, popularly known as *aonla* belongs to the family Euphorbiaceae and is believed to have originated in the central and southern parts of India. The country ranks first in the area and production of this fruit (Priya & Khatkar 2013). It is a very rich source of vitamin C and phenolic compounds, two potent antioxidants. The vitamin C content of this fruit ranges from 450 to 600 mg100g ^− 1^, which is almost 10 times higher than in citrus fruits, a content which is rarely seen in fresh fruits. The leucoanthocyanins in the fruit along with the high amounts of phenolics is considered to provide stability to the ascorbic acid content. Indian gooseberry is widely used in traditional forms of medicines like Ayurveda and Unani to treat diseases like diabetes, jaundice, dyspepsia, diarrhea, scurvy, bronchitis, skin diseases, various types of cancers and the like. The bioactivity of Indian gooseberry fruit extract in its anticancer and antitumour functions are thought to be imparted by the high levels of phenolics and flavonoids. Tannins, a group of phenolic compounds from Indian gooseberry fruit extract identified by HPLC method and having anticancer properties are ellagic acid, corilagin, pyrogallol, chebulagic acid, gallic acid and also, the flavonoid, quercetin (Zhao et al. 2015). Due to the high acidity as well as the very high astringency imparted by phenolic compounds, the fruit is seldom relished in its fresh form. However, there exists ample opportunities to transform it into numerous value added products.

Curcumin, a bright yellow to orange coloured compound is indispensable in many vegetable preparations as well as in non-vegetarian dishes. Addition of curcumin to dishes either in powder form or as oleoresin drops makes food items appealing and thereby, increases the marketability of the product. Turmeric (*Curcuma longa*) is a rhizomatous herbal spice native to India, belonging to the Zingiberaceae family and is valued for the polyphenol, curcumin which has enormous applications in food as well as pharmaceutical industries. Curcumin, also known as diferuloylmethane is chemically 1, 7-bis (4-hydroxy-3-methoxyphenyl)-1, 6-heptadiene-3, 5-dione (Hewlings & Kalman 2017). Curcumin has been found to have anti-inflammatory properties, alleviation of metabolic disorders, relief from pain, improvement of eye sight and may also help in the functioning of kidneys. Turmeric / curcumin is used in different parts of the world for various purposes. In India, it is an indispensable ingredient in curries as well as in majority of vegetarian and non-vegetarian dishes. Different forms of curcumin are available in the market such as capsules, ointments, powder, soaps, tablets etc. (Moghadamtousi et al. 2014). The United States Food and Drug Administration has approved curcuminoids as “Generally Recognised As Safe”, more popularly known as ‘GRAS’ compounds (Gupta et al. 2013). Three curcuminoids viz. curcumin, demethoxycurcumin and bisdemethoxycurcumin in clinical trials have been found to have good tolerability, of doses up to 12,000 mg/ day (Lao et al. 2006). Curcumin has established antioxidant activity as evident from its efficacy on oxidative stress indicators such as plasma activities of superoxide dismutase, catalase and also, in serum concentrations of glutathione peroxidase as well as lipid peroxides (Sahebkar et al. 2015).

The compound piperine is unique in its culinary properties as the characteristic hot and pungent flavour of the compound cannot be substituted by any other spice and therefore, it is widely used in many dishes across countries. Black pepper (*Piper nigrum* L.) is a member of the Piperaceae family, which is cultivated mainly in the humid tropics, is widely consumed across the globe owing to its pungent principle, an alkaloid ‘piperine’. Piperine is chemically 1-peperoyl piperidine. Several forms of black pepper are traded in the market which include dehydrated black pepper berries, dehydrated green berries, green berries in brine, dehydrated white pepper from which the pericarp is removed, oleoresin and essential oil. These diverse processed products are indispensable ingredients in many non-vegetarian preparations in the tropics. The compound ‘piperine’ possesses innumerable pharmacological properties like antioxidant, antipyretic, antihypertensive, anti-inflammatory, anti-microbial and immunomodulatory properties. In spite of its use as an indispensable ingredient in many culinary preparations, it also finds place in several Ayurvedic medicines. Besides its medicinal use, it is also used as a preservative and also, used in perfumery (Damanhouri & Ahmad 2014). Piperine is known to enhance bioavailability of many nutrients and drugs by interfering in metabolic pathways. This property of piperine is being utilized in the preparation of several drugs aimed at alleviating or even curing many metabolic disorders. Antioxidant activity of three Piper species viz. *Piper nigrum*, *P. guineense* and *P. umbellatum* on hamsters fed with atherogenic diet at the rate of 1 g/kg and 0.25 g/ kg for 12 weeks resulted in antioxidant protective activity against oxidative stress occurring in cardiac, hepatic and renal tissues (Agbor et al. 2012).

Flavouring food items with ginger either in the fresh or processed form, imparts a pungency characterized by its sharp flavour. Ginger (*Zingiber officinale* Roscoe.) belongs to the Zingiberaceae family, same as that of turmeric, is a widely consumed condiment either as fresh or dried rhizomes or in its processed forms like oleoresin and essential oil. It is used in a variety of dishes due to the pungency attributed by the ketone, gingerol, the primary ingredient responsible for its unique flavour. Gingerol is chemically 1-(4′-hydroxy-3′- methoxyphenyl)-5-hydroxy-3-decanone (Bode & Dong 2011). Ginger has been used since time immemorial to cure or manage intestinal problems, common cold, cough, fever etc. A clinical trial involving humans were given ginger at doses ranging from 100 mg to 2.0 g as a single oral dose and the findings revealed that free forms of gingerol and shogaol could not be detected; instead the glucuronide of these compounds were detected in the conjugated form (Zick et al. 2008). Ginger is well known for its antioxidant properties and has been found to retard oxidative stress related to ageing process. A very high level of total antioxidants (3.85 mmols/100 g) was reported in ginger rhizomes which was next only to pomegranate and some berry fruits (Halvorsen et al. 2002).

Fruity flavour contributed by the unique sugar-acid blend is typical of citrus fruits and probably, this might be the reason for their large-scale cultivation and extensive consumption across various parts of the globe. Acid lime (*Citrus aurantifolia* L.), a member of the citrus group of fruits is believed to have originated in India and therefore, it is widely cultivated in the tropical and subtropical parts of the country. The fruit is well known for its vitamin C content, a potent antioxidant and is therefore, widely used in traditional medicines and also, for preparation of various types of fruit based drinks. Owing to its vitamin C content, the fruit is known to maintain oral health by reducing the incidence of gingivitis and scurvy. The fruit is well known for its role in alleviating many gastric disorders like indigestion, peptic ulcers and constipation (Ganguly 2014).

## Materials and methods

### Preparation of functional herbal drink

Fresh, sound Indian gooseberry fruits were procured from the local market, followed by washing in plain tap water. The cleaned fruits were then crushed in a fruit crusher along with the seeds. The crushed mass was then taken in a muslin cloth bag, followed by extraction of juice in a stainless steel basket press. As Indian gooseberry juice as such is highly acidic and astringent in nature, in order to improve its palatability, it was reconstituted with other ingredients like turmeric, ginger, acid lime juice, black pepper and the total soluble solids was raised to 13 ^0^ Brix by adding sugar syrup. Freshly extracted Indian gooseberry juice amounting to 20% (v/v) was added to sugar syrup containing 5% ginger juice (v/v) extracted from freshly harvested rhizomes, 2% each of turmeric rhizome powder and black pepper powder (w/v). Upon cooling down of the sugar syrup thus prepared, was added with 5% acid lime juice of 2.45% of titratable citric acid content in order to impart a fruity flavour to the product. The entire mixture containing water, Indian gooseberry juice, acid lime juice, turmeric rhizome powder, ginger rhizome juice and sugar were subjected to homogenization in a two-stage stainless steel homogenizer with an operating pressure of 175 Bar (2500 psi) with a speed of 235 SPM. The homogenized herbal drink thus prepared was filled into 500 ml capacity glass bottles followed by screw capping with a metal cap and was subsequently pasteurized at 100 ^0^ C for 10 minutes and these bottles were allowed to cool down to room temperature. The pasteurized herbal drink in glass bottles were subsequently stored at a temperature of 5 ± 2 ^0^ C. Changes in phytochemical constituents and antioxidant activity was determined initially as well as at monthly intervals for a period of 3 months. The experiment was repeated thrice in order to confirm the findings of the experiment.

### Phytochemical analysis and determination of antioxidant activity

#### Phytochemical analysis

Changes in the quality of the product, especially phytochemical constitution and antioxidant activity were done as per standard procedures. Determination of pH values of the product was done using a digital pH meter (Scientific Tech, S 72025, India). TSS values were measured using a digital refractometer (Atago, Pal 1 & 2, Japan) and the values were expressed in ^0^ Brix. Ascorbic acid content of the product was determined by titrating a known weight of the sample containing 3% meta phosphoric acid with 2, 6-dichlorophenol indophenol dye. The content of ascorbic acid (vitamin C) was expressed in mg 100 g^− 1^ (AOAC 1998). Total carotenoids were extracted with acetone and petroleum ether in a separating funnel and 3% acetone in petroleum ether formed the blank (Ranganna 1997). To absorb excess water, anhydrous sodium sulphate was used during extraction and finally, the optical density values (452 nm) were found out using a UV-Visible spectrophotometer (Model No. 1800, Shimadzu, Japan). Determination of total flavonoids was done as per the method of Chang et al. (2002). Sample was extracted with ethanol, followed by addition of 1.5 mL of methanol, 10% aluminium chloride (0.1 mL), 1 M potassium acetate (0.1 mL), along with 2.8 mL of distilled water and was finally kept for 30 minutes at room temperature. Optical density values of the reaction mixture were measured at 415 nm in the UV-Visible spectrophotometer. Total phenolics in the product were estimated using Folin- Ciocalteau reagent as described by Asami et al. (2003). Five milliliters of sample was added to 1 mL of 80% ethanol, followed by addition of 0.3 mL of Folin-Ciocalteau reagent. To this, 10 mL of 7% sodium carbonate solution was added after 6 minutes, mixed thoroughly and was left for 2 h. Reaction of phenols and phosphomolybdic acid in alkaline medium resulted in a blue coloured complex, followed by reading the absorbance values at 740 nm (UV-Visible spectrophotometer), Model No. 1800, Shimadzu, Japan. Total phenolics were quantified using a calibration curve. Gallic acid formed the standard for phenolics estimation. Curcumin content in the product was measured as per the method suggested by Ranganna (1997). A known weight of the sample was dissolved in 250 mL of absolute ethanol, followed by refluxing the contents for 3 to 5 h. The extract was decanted into a volumetric flask. One to two milliliters of suitable aliquot was diluted to 10 mL with absolute alcohol and the absorbance was measured at 425 nm in a UV-Visible spectrophotometer. Curcumin content was calculated using the formula, 0.0025 x A_425_ x volume made up x dilution factor × 100 / 0.42 x weight of sample × 1000, since 0.42 absorbance at 425 nm is equal to 0.0025 g curcumin.

#### Antioxidant activity

Radical scavenging potential of the product was determined by three assays, viz. DPPH (2, 2-diphenyl − 1-picrylhydrazyl) radical scavenging activity, FRAP (ferric reducing antioxidant power) and ABTS (2, 2′-azino-bis (3-ethylbenzothiazoline-6-sulfonic acid). DPPH method of radical scavenging potential was determined as per the method of Braca et al. (2001). Methanol extract of the sample was added with 1.3 mL DPPH, followed by reading the values of the reaction mixture at 517 nm. Radical scavenging percentage was calculated from the formula, % inhibition of radical = (Control-Sample × 100) / Control. Gallic acid was used as the standard and the concentration of sample providing 50% radical inhibition (IC _50_ values) were calculated from the formula given above and were expressed in μg ml^− 1^. Antioxidant potential of the sample by FRAP assay is based on the reduction of Fe3+ tripiridyltriazine (colourless) to Fe2+ tripiridyltriazine (blue coloured complex) due to the reaction of electrons donated by antioxidant compounds (Benzie et al 1999). The reaction was monitored by observing the change in absorbance at 593 nm. FRAP reagent was prepared by combining the compounds 300 mM acetate buffer, 10 mL tripiridyltriazine in 40 mM hydrochloric acid along with 20 mM ferric chloride (FeCl36H2O) in the ratio of 10:1:1 at 37 ^0^ C. 3.995 ml of FRAP reagent was mixed with 5 μL of the product, followed by thorough mixing. The reaction mixture resulted in an intense blue coloured complex due to the reduction of ferric tripiridyltriazine to ferrous tripiridyltriazine form. The absorbance was recorded at 593 nm against a reagent blank consisting of 3.995 mL FRAP reagent and 5 μL distilled water, after incubation for 30 minutes at 37 ^0^ C. Calibration curve was prepared using different concentrations of FeSO4 by plotting the absorbance at 593 nm. Ascorbic acid was used as standard in this assay. Antioxidant capacity of the product based on ABTS assay was assessed by ABTS radical cation decolourisation (Re et al. 1999). The reaction between 7 mM ABTS dissolved in water and 2.45 mM potassium persulphate in 1: 1 ratio produced ABTS^.+^ cation radical, which was subsequently kept in a dark room for 12 to 16 hours before analysis. Methanol was added to ABTS^.+^ solution in order to obtain absorbance of 0.700 at 734 nm. Five microliters of the product was added to 3.995 mL of ABTS^.+^ solution and after 30 minutes, the absorbance was recorded. Per cent inhibition was obtained from the formula, ABTS^.+^ scavenging effect (%) = (AB-AA)/AB × 100. AB is absorbance of ABTS radical along with methanol, AA is absorbance of ABTS radical in combination with sample extract/ standard. The sample concentration providing 50% inhibition (IC _50_) was determined from the graph of inhibition percentage against the concentration.

Statistical analysis of the data obtained in the present study was done using Completely Randomised Design consisting of five replications and is presented as mean values ± standard deviation as per Web Based Agricultural Statistics Software Package (WASP). The mean values were compared at a confidence level of 95% (*p* = 0.05) using Duncan’s multiple range test. The experiment was repeated thrice.

## Results and discussion

Changes in phytochemical constituents and antioxidant capacity of the product during storage are presented as follows.

### Phytochemical constitution

#### pH

Shelf life of food, to a very great extent is determined by its pH content. Foods with low pH are known to have higher shelf life. Besides, the type of microorganism as well as the rate at which they multiply are also dependent on the pH content as pH denotes the negative logarithm of H+ concentration. In high- acid foods like fruits and their products, the pH tend to be in the range of 3 to 4. The stability of many phytochemicals in fruits and their products to a very great extent is dependent on the pH of these products. The initial pH of the herbal drink was 2.90. This low pH may be due to the naturally high acid content of fruit juice in Indian gooseberry as well as in acid lime fruits. A pH of 3.08 ± 0.08 was reported by Thakur et al. (2018) in syrup prepared from wild *aonla* (Indian gooseberry) fruits. Similarly, acid lime fruits also have an acidity level of more than 2.0%. pH of the product showed a declining trend throughout storage (Table 1). The decline was significantly influenced by the storage period. Though high acid drinks are not favoured by consumers, an optimum blend of sugar-acid will considerably improve the flavour of the product. pH of the product was 2.85, 2.76 and 2.70 after first, second and third months of storage, respectively. Decline in pH of the product might have been due to the rise in acidity as a result of pectinic acid and also, due to fermentation of sugars due to metabolic activities of microbes during storage which might have produced organic acids in the product during storage. Drop in pH of passion fruit juice from 3.39 to 3.20%, 3.96 to 3.86 in pineapple juice and that of mango juice from 4.19 to 3.90% was reported by Kaddumukasa et al. (2017) when these fruit juices were stored at 4 ^0^ *C.* Silva et al. (2015) observed a pH of 3.86% in pineapple pulp stored under refrigerated conditions (4 ^0^ C) in dark bottles.

**Table 1:** pH, TSS (^o^ Brix) and ascorbic acid content (mg/100 g) of functional herbal drink during refrigerated storage

Storage period	pH	TSS (^**o**^Brix)	Ascorbic acid (mg/100 g)
**Initial**	2.90^a^	13.20^a^	61.00^a^
**1 MAS**	2.85^b^	12.30^b^	54.47^b^
**2 MAS**	2.76^c^	11.60^c^	42.36^c^
**3 MAS**	2.70^d^	11.40^c^	36.39^d^
**CD value (5%)**	0.026	0.385	2.671
**SE (± m)**	0.009	0.128	0.891
**SE (± d)**	0.012	0.182	1.26

*MAS* Months after storage; a, b, c denote significant difference between treatments

#### Total soluble solids (TSS)

Water soluble substances like sugars, organic acids, some proteins, water soluble vitamins like ascorbic acid etc. constitute the total soluble solids in foods. TSS content is an indication of consumer acceptability of fresh as well as processed food items. A higher TSS content is positively correlated to higher acceptability by consumers. In fruits and fruit based drinks, an optimum blend of sugar and acid is more preferred to higher sugar content or acidity levels. Besides, higher TSS contents in fruit products impart greater stability to phytochemicals in such products. Indian gooseberry based herbal drink had an initial TSS content of 13.20 ^0^ Brix (Table 1). This level of TSS in the product was due to the reconstitution of Indian gooseberry juice with sugar syrup which resulted in a higher TSS content in the product compared to fresh fruits, which normally have a lower TSS content (around 8 ^0^ Brix). This level of TSS was maintained in order to slightly mask the originally higher levels of astringency and acidity seen in the fruits. Total soluble solids declined significantly during the first 2 months of storage, which were 12.30 and 11.60 ^0^ Brix, respectively. However, after 3 months of storage, the decline was insignificant (11. 40 ^0^ Brix). Decline in TSS content of the product may be due to fermentation process during which sugars are broken down into ethanol, carbon dioxide and water, along with production of volatile compounds. This finding is in agreement with those reported by Saji Gomez et al. (2022) wherein a drop in TSS was recorded in Indian blackberry nectar during storage. Kaddumukasa et al. (2017) also reported decline in ^0^ Brix values in fresh unpasteurized juice of fruits like mango, pineapple and passion fruit during a storage period of 12 days under ambient and refrigerated conditions. However, the findings with regard to TSS content in the present study contradicts the one reported by Thakur et al. (2018) who noticed an increase in TSS content of syrup prepared from wild *aonla* (Indian gooseberry) fruits. Sandi et al. (2004) observed that there was a reduction in non-reducing sugar (sucrose) in the juice of yellow passion fruit when held for 120 days under both ambient (25 ± 5 ^0^ C) and refrigerated (5 ± 1 ^0^ C).

#### Ascorbic acid

Plant foods, particularly fruits and vegetables are primary sources of vitamin C, which is chemically known as ascorbic acid. It is one of the important antioxidants found in abundance in many fruits and vegetables and hence, these food items are indispensable as components in a balanced diet. All the more important is the fact that humans cannot synthesize this vitamin, it has to be taken in through consumption of fruits and vegetables. Moreover, inclusion of vitamin C in adequate amounts will benefit humans in warding off many diseases related to metabolism and immunity and also, will improve the overall well-being of people. It has been reported to be highly bio-available and is also soluble in water. The Indian Council of Medical Research recommends a dietary allowance of 90 and 75 mg of vitamin C for adult men and women, respectively. The initial ascorbic acid content of the herbal drink was 61.0 mg100g^− 1^ (Table 1). The drop in ascorbic acid content of the product might be due to its degradation during pasteurization as vitamin C is highly thermo-labile. Loss in vitamin C content could happen due to peroxides formed during thermal processing as well as due to light exposure and presence of enzymes like ascorbate oxidase and peroxidase can accelerate the degradation process. An ascorbic acid content of 180. 71 mg100g^− 1^ was recorded in wild *aonla* syrup (Thakur et al. 2018), which is a concentrated product that needs dilution before serving. Unlike fruit syrup, the herbal drink has to be consumed in the original form in which it is available. Ascorbic acid content showed significant decline in the product throughout storage. The herbal drink contained 54.47, 42.36 and 36.39 mg100g^− 1^ after first, second and third months of storage, respectively. Fall in vitamin C content of the product might be due to its degradation to dehydro-ascorbic acid or due to its conversion into furfural during storage. Ascorbic acid is highly sensitive to photo-oxidation. Thakur et al. (2018) also reported that the ascorbic acid content of wild Indian gooseberry fruit syrup declined during storage. The results are in accordance with those already reported by Hosseini et al. (2015) in orange nectar prepared with steviol glycosides and also the decline of ascorbic acid in Indian blackberry nectar during storage by Gomez et al. (2022). Chia et al. (2012) reported that the presence of dissolved oxygen in fruit juices could lead to its degradation during long-term storage. Further, storage temperature of the product, its method of processing as well as the type of packaging container can all affect the retention of vitamin C. Fruit juices retaining 50% of their initial ascorbic acid content could be treated as unmarketable or even termination of their shelf life (Chia et al. 2012). In the present study, vitamin C of the herbal drink under refrigerated storage fell from 61.0 to 36. 39 mg100g^− 1^, even after 3 months of storage which is an indication of its shelf life.

#### Total phenolic content

Phenolics are potent antioxidant compounds which are found in plenty in many plant based foods. There are several types of phenolic compounds and the type as well as the concentration vary with fruit or vegetable and the stages of maturity. In general, raw or immature fruits will have higher total phenolics and their level decreases with advancement in maturity and ripening. Phenolic compounds also have direct bearing on flavour of fruits, particularly on the taste of the fruit. Fruits with higher levels of phenolics in general tend to be acrid or astringent in taste. Indian gooseberry is one of the richest sources of phenolic compounds, which make the fruit highly astringent. The initial total phenolic content was 184.0 mg100g^− 1^ (Table 2). Tannins are the major phenolic compounds in Indian gooseberry fruits. The various tannins identified in Indian gooseberry fruits by HPLC method are ellagic acid, corilagin, pyrogallol, chebulagic acid and gallic acid (Zhao et al. 2015). Curcumin is the major polyphenol found in the rhizomes of turmeric. Curcumin is also known as diferuloylmethane which is chemically 1,7- bis (4-hydroxy-3-methoxyphenyl)-1, 6-heptadiene-3, 5-dione (Hewlings & Kalman 2017). About 32% reduction in total phenolic compounds was recorded in pasteurized (90 ^0^ C for 30 seconds) apple juice by Aguilar-Rosas et al. (2007) in comparison with the untreated sample. In contrast, Thakur et al. (2018) reported a higher value of 549.0 ± 0.48 mg100g^− 1^ in freshly prepared wild Indian gooseberry fruit syrup, which was not heat processed. Reduction in total phenolic content might have occurred due to thermal and oxidative changes during preparation and subsequent heat treatment of the product as a result of polymerization of phenolics. There was significant increase in total phenolic content of the herbal drink throughout storage. Total phenolic content rose from 184.0 to 225.63, 307.84 and 463.79 mg100g^− 1^ after first, second and third months of storage, respectively. The finding is in conformity with the one reported in Indian blackberry nectar during storage by Saji Gomez et al. (2022). The present findings also corroborates the one observed by Piljac-Zegarac et al. (2009) in six dark coloured juices wherein the total phenolics rose during 29 days of refrigerated storage. Canning of raspberries and blueberries at 100 ^0^ C for 28 minutes and 100 ^0^ C for 22 minutes, respectively increased the phenolic content by 50% (Sablani et al. 2010). However, the present findings contradict the results of Thakur et al. (2018) who reported fall in the level of phenolic compounds in wild Indian gooseberry syrup stored under ambient as well as refrigerated conditions for 6 months.

**Table 2:** Total phenolics, flavonoids, curcumin and total carotenoid content (mg/100 g) of functional herbal drink during refrigerated storage

Storage period	Total phenolics (mg/100 g)	Flavonoids (mg/100 g)	Curcumin (mg/100 g)	Total carotenoids (mg/100 g)
**Initial**	184.00^a^	153.00^a^	31.00^a^	119.98^a^
**1 MAS**	225.63^b^	134.06^b^	23.70^b^	110.39^b^
**2 MAS**	307.84^c^	119.69^c^	14.89^c^	94.58^c^
**3 MAS**	463.79^d^	106.00^d^	13.02^c^	77.86^d^
**CD value (5%)**	17.808	5.360	2.350	3.663
**SE (± m)**	5.940	1.788	0.784	1.222
**SE (± d)**	8.4	2.528	1.109	1.728

*MAS* months after storage; a, b, c denote significant difference between treatments

#### Total flavonoids

Flavonoids are group of phenolic compounds having antioxidant, antiviral, antibacterial and anticancer properties. In general, its structure is that of a two benzene rings namely, A and B linked by three carbons in the oxygenated heterocyclic C ring. Several types of flavonoids are seen in fruits in the esterified or in conjugated forms in many fruits and these include flavones, flavonols, flavonones etc. depending on the plant. Owing to these bioactive properties, flavonoids are well recognized as vital components of a healthy diet with numerous health protective properties. The initial total flavonoid content of the herbal drink was 153.0 mg 100 g^− 1^ (Table 2). Thermal processing of fruits is associated with different forms of media like water and air for transfer of energy. Since Indian gooseberry, turmeric, ginger and acid lime are good sources of phenolic compounds, the herbal drink prepared utilizing them may have resulted in the high total flavonoid content in the drink. Jiratanan and Liu (2004) reported that table beets when heat processed at 105 to 125 ^0^ C for 15 to 45 minutes, the total, bound and free forms of phenolics and flavonoids were either retained or increased. Total flavonoid content of the herbal drink fell significantly during storage. From 153.0 mg100g^− 1^ of its initial level, the content fell to 134.06, 119.69 and 106.0 mg100g^− 1^ after first, second and third months of storage. According to Balunkeswar et al. (2015), storage can be considered as passive processing even though no form of energy is applied directly to food items during storage. Temperature variations and enzymatic reactions during storage can lead to degradation of phenolic compounds, particularly flavonoids, during storage. Enzymes like polyphenol oxidase, peroxidase and glycosidase along with photo oxidation can lead to destruction of flavonoids during storage. Ortho-dihydrophenols are oxidized to ortho-quinones as a result of catalysis by the enzyme polyphenol oxidase, turning them into polymers which are brown in colour and subsequently leading to browning or darkening of the product during storage. Destruction of phenolic compounds as a result of oxidation can occur during prolonged storage as well as due to intrinsic properties of food matrix pH, availability of oxygen, water activity, soluble solids and temperature (Nicoli et al. 1999). Anthocyanins and other flavonoids are known to have low stability and can be degraded as a result of covalent and non-covalent interactions when they react with other compounds. Besides, pH, light, heat and oxygen can also affect their stability in fruit juice (Zhang et al. 2019).

#### Total carotenoids

Carotenoids are C-40 fat soluble compounds having eight isopentenyl pyrophosphate units and occur in hues of yellow, red and orange colours in plant organs. Lutein, zeaxanthin, lycopene, xanthophyll and β-carotene are some of the carotenoids of dietary significance found commonly in fruits and vegetables. They have a wide range of health protective properties such as antioxidant, anticancerous, anti-inflammatory and can also contribute to the general well-being of humans which include slowing down of macular degeneration as well. Owing to the numerous health benefits of carotenoids, they are considered indispensable compounds in healthy and balanced diets. Initial total carotenoid content of the herbal drink was 119.98 mg100g^− 1^ (Table 2). The high carotenoid content of the product might be due to the incorporation of turmeric in the preparation of the drink. Thermal processing might have resulted in the disconnection of carotenoids from turmeric and subsequently got extracted into the product. Total carotenoids decreased significantly during storage. From an initial level of 119.98 mg100g^− 1^, it declined to 110.39, 94.58 and 77.86 mg100g^− 1^ after first, second and third months of storage. Degradation of carotenoids during storage may be mainly due to photo-oxidation. As the herbal drink was stored at relatively lower temperature (5 ± 2 °C), chances of thermal degradation are less likely during storage. Further, carotenoids can be converted into several forms of isomers which may also indicate the extent of degradation. Moreover, loss of colour intensity of the product is direct indication of degradation of carotenoids. Lu et al. (2019) studied carotenoid degradation in sterilized ‘Cara Cara’ (a bud mutant of *Citrus sinensis*) juice over a storage period of 16 weeks at 4, 20, 30 and 40 ^0^ C. They reported slight degradation of carotenoids at all storage temperatures but the decline was not significant. They opined that bi-exponential (both irreversible and reversible) degradation of carotenoid occurred during storage. In irreversible degradation, carotenoids were degraded into volatiles or epoxides whereas in reversible degradation, isomerization of carotenoid was involved. Castro-Lopez et al. (2016) studied fluctuations in total carotenoid content of eight fruit beverages over a storage period of 20 days. They reported a 25% decline in carotenoids of juices at 4, 8 and 11 ^0^ C, during the first 12 days. In some juices, a 50% decline was noticed after the end of the storage period. The loss in carotenoid during storage was mainly attributed to oxidation induced by oxygen, metal ions, light, peroxides, enzymes and also due to the geometric isomerization of the polyene chain of carotenoids.

#### Curcumin content

Curcumin is a phenolic compound obtained from the rhizomatous roots of turmeric plant which are cleaned, washed and dried in order to obtain turmeric powder having enormous health benefits and also, an important ingredient in many culinary preparations. Curcumin’s health benefits include properties like antioxidant, anti-diabetic, anti-inflammatory and anti-cancer activities that have been proved in several clinical trials. The yellow to orange colour imparted by curcumin is widely used to produce functional beverages as well in order to improve the appearance of such products and will thus enhance the marketability of these products by making the product look bright and attractive. As curcumin has poor solubility in water, black pepper, a source of piperine was added to the herbal drink in order to enhance the bioavailability of curcumin. Delecroix et al. (2017) reported that consumption of a diet supplemented with 2 g of curcumin and 20 g of piperine was helpful in alleviating muscle soreness in rugby players after grueling practice sessions. Numerous health benefits have been reported for curcumin and these benefits are primarily due to the anti-inflammatory and anti-oxidant properties (Hewlings & Kalman 2017). The initial curcumin content of the herbal drink was 31.0 mg100g^− 1^ (Table 2). Curcumin compares well in terms of safety aspects. According to the Joint United Nations and World Health Organisation Expert Committee on Food Additives (JECFA) and European Food Safety Authority (EFSA), the ADI (Allowable Daily Intake) of curcumin is in the range of 0 to 3 mg/ kg body weight. Total curcumin content of the product showed a declining trend during storage. Curcumin content of the herbal drink declined significantly after first and second months of storage wherein the levels remained at 23.70 and 14.89 mg100g^− 1^, respectively. However, the content was insignificant after the third month of storage, which was 13.02 mg100g^− 1^. Degradation of curcumin can happen through photo degradation and also, through solvolysis (Nelson et al. 2017). In solvolysis, nucleophilic elimination or substitution occurs as a result of interaction with solvent molecules. The attack on nucleophile happens on α, β-unsaturated ketone part of curcumin. Solvolysis of heptadienone chain in aqueous alkaline buffer can degrade up to 90% of curcumin to produce ferulic acid, vanillin, ferulic aldehyde, vanillic acid etc. (Priyadarsini 2014). Auto-oxidation of curcumin can happen as a result of oxygen incorporation through a radical chain reaction which results in the production of a bicyclopentadione. Findings of the present study is in accordance with those reported by Dwiloka et al. (2019) wherein the curcumin content of brewed spiced drink containing turmeric and ginger, fell from 46.916 ppm in the first brewing to 16.184 ppm in the second brewing, indicating an overall decrease of curcumin content by 30.732 ppm. Curcumin is light-sensitive, in both solid and liquid forms and therefore, it is advisable to protect samples/ products containing curcumin from exposure to light (Kotha & Luthria 2019).

#### Antioxidant activity

**Fig. 1:**
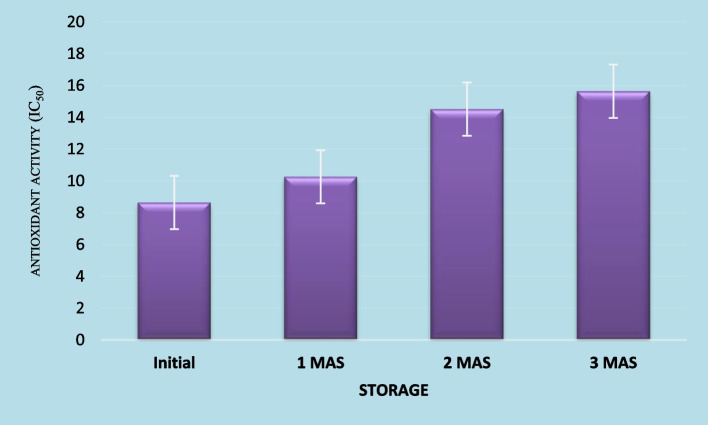
Antioxidant activity (IC_50_ μg/ml) of functional herbal drink during refrigerated storage by using ABTS assay

**Fig. 2:**
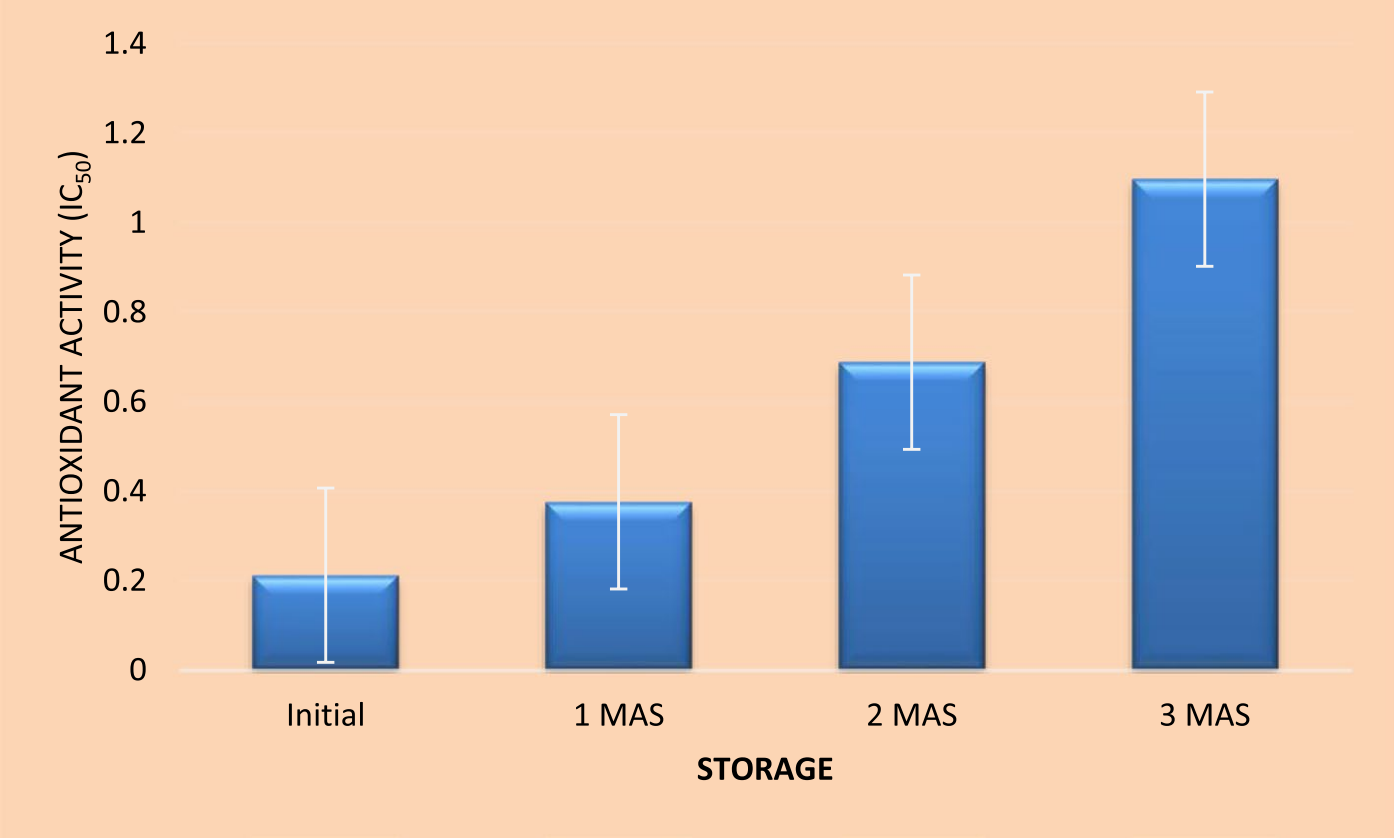
Antioxidant activity (IC_50_ μg/ml) of functional herbal drink during refrigerated storage by using DPPH assay

**Fig. 3:**
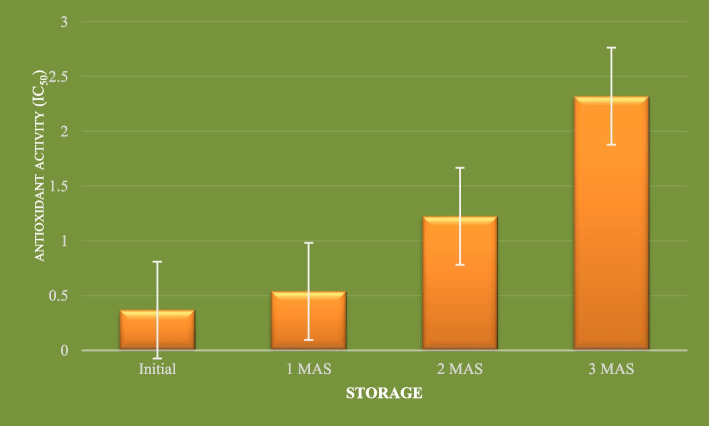
Antioxidant activity (IC_50_ μg/ml) of functional herbal drink during refrigerated storage by using FRAP assay

Phytochemicals like phenolic compounds, ascorbic acid, carotenoids, vitamin E etc. have strong antioxidant properties owing to their capacity to quench free radicals like superoxide, peroxides, hydrogen peroxide, hypochlorite, hydroxyl radical, nitric oxide radical, superoxide anion radical etc. Free radicals are characterized by unpaired electrons in their atomic orbits which render them highly reactive and are responsible for many degenerative diseases like cancer, arthritis, premature ageing, atherosclerosis, damage of cellular components like DNA, lipids, protein and will also lead to reduction in immunity levels etc. They are either produced in the body as a result of normal metabolic activities or due to exposure to harmful radiations like UV rays, x-rays and ozone. Disturbances in the balance of generation of free radicals and antioxidants will lead to oxidative stress and in order to maintain this balance, supply of antioxidants through diet assumes significance. Fruits, vegetables and their products are important sources of antioxidants. Role of antioxidants is to donate an electron as a result of which the free radicals are neutralized and thereby, the occurrence of many degenerative diseases can be either delayed or prevented. Antioxidant activity of the herbal drink was determined by three assays, viz. ABTS, DDPH and FRAP. The initial IC _50_ values of the herbal drink by ABTS, DPPH and FRAP assays were 8.64, 0.212 and 0.368 μgml^− 1^, respectively (Figs. 1, 2 and 3). A lower IC _50_ value is an indication of higher antioxidant potential of the product. The values indicate that developed herbal drink has greater antioxidant capacities. This might be due to the raw materials used in the preparation of the product. Indian gooseberry is one of the richest sources of vitamin C and polyphenols, two potent antioxidant compounds. Kaur and Kapoor (2005) evaluated the antioxidant activity of 19 fruits consumed as part of Indian diet. They evaluated the antioxidant activity by FRAP, β-carotene lineolate and super-oxide anion scavenging activity assays. Among the 19 fruits evaluated, which included fruits like Indian gooseberry (*Emblica officinalis*) pomegranate, strawberry, plums, Indian blackberry (*Syzigium cumini*), black grapes, *phalsa (Grewia subinequalis)*, apple, *ber (Syzigium jujube)*, *bael* (*Aegle marmelos*), sapota, papaya, banana and guava, Indian gooseberry had the highest total phenolics content (290 mg100g^− 1^) and highest antioxidant content (56.8 mM by FRAP assay, 92% by β-carotene lineolate assay and 85% super-oxide anion scavenging activity). Similarly, addition of spices and condiments like turmeric, black pepper and ginger might have compounded the antioxidant capacity of the product as curcumin in turmeric, gingerol in ginger and piperine in black pepper are compound with proven antioxidant and anti-inflammatory properties. Moreover, thermal processing might have resulted in the release of free phenolic acids from the bound form of phenolic compounds present in the raw materials used which might have supplemented the antioxidant potential of the herbal drink. Garg and Ahuja (2015) reported that a functional herbal summer drink with *bael* and Indian gooseberry fruits as major ingredients could scavenge 75% free radicals when determined by DPPH assay alone. Comparison of antioxidant capacity of fruit juices of apple, mandarin orange, persimmon and pear in Korea by Pyo et al. (2014) revealed that the antioxidant capacities determined by DPPH and FRAP assays strongly corresponded with the total phenolics and ascorbic acid contents. They also found that in all the four fruit juices studied, a positive linear correlation existed between ascorbic acid levels and the antioxidant capacities measured by FRAP and DPPH assays which had ‘r’ values 0.8901 and 0.9115, respectively. Findings of the present study corroborates those reported by Thaipong et al. (2006) wherein the mean antioxidant activity values of guava fruit extracts determined by ABTS, DPPH and FRAP assays were 31.1, 25.2 and 26.1 μM TE/g, respectively. Determination of antioxidant activity of the herbal drink by ABTS, DPPH and FRAP assays showed significant decline throughout the storage period. The radical scavenging activity determined by ABTS assay decreased to 10.256, 14.510 and 15.637 μgml^− 1^ after first, second and third months after storage, respectively. Similarly, the radical scavenging activity by DPPH method reduced to 0.376, 0.668 and 1.097 μgml^− 1^ after first, second and third months after storage, respectively. In FRAP assay also, the radical scavenging activity declined to 0.539, 1.224 and 2.321 μgml^− 1^ from the initial value of 0.368 μgml^− 1^ after the first, second and third months of storage, respectively. The decline in radical scavenging activity might be due to degradation of antioxidant compounds in the product like ascorbic acid, carotenoids, flavonoids and curcumin. However, the total phenolics, another group of antioxidant compounds in the herbal drink showed an upward trend during storage. This might be due to the conversion of polyphenols into free phenolic acids that got extracted into the drink and the reaction might have continued during storage. Further, heat processing might have inactivated the enzyme polyphenol oxidase, which is responsible for degradation of phenolic compounds. This result is in accordance with the one reported by Zhang et al. (2019) who observed that heat processing of blueberry juice enabled increased extraction of phenolic compounds. However, the same authors reported decline in total phenolics content of blueberry juice during storage at 40 ^0^ C for 10 days. Therefore, findings of the present study indicate that storage of herbal drink at low temperature might have prevented deterioration of phenolic compounds. Piljac-Zegarac et al. (2009) evaluated changes in total phenolics and antioxidant activity of six dark coloured fruit juices during storage under refrigeration. They reported that the antioxidant activity of these fruit juices as determined by DPPH assay and cyclic voltammetry methods revealed significant decline in radical scavenging activity in five out of six juices studied and also, all the juice from all six fruits showed a significant decline in Trolox equivalent antioxidant capacity over a period of 29 days under refrigerated storage. A significant linear correlation was seen between values determined by both cyclic voltammetry and DPPH radical scavenging assay with a r^2^ value of 0.62. However, the findings of the present study contradicts the results reported by Garg and Ahuja (2015) wherein no significant decline in antioxidant activity was recorded in functional herbal summer drink containing *bael* fruit juice as the major ingredient (80% v/v) along with Indian gooseberry (15% v/v), over a period of three-month storage when determined by DPPH assay alone.

## Conclusions

Incidence of covid-19 pandemic has reinforced the essentiality of consuming healthy diets. Foods rich in antioxidants, particularly of fruit and vegetable origin are easily available, relatively cheaper but are of immense health or medical benefits to maintain higher levels of immunity and thereby alleviating the seriousness or complications arising as a result of such diseases. Development of herbal functional beverages offer ample scope to the food industry in order to satisfy the growing demands of consumers to have healthy, natural and ‘chemical-free’ food items.

Fruits, vegetables, spices and condiments are viable alternatives to synthetic drugs as they are loaded with health protective secondary metabolites. The herbal functional beverage developed from Indian gooseberry fruit juice in which spices and condiments are incorporated, can be considered as a health drink having good amounts of phytochemicals like ascorbic acid, total phenolics, flavonoids, total carotenoids and curcumin, all of which have immense antioxidant properties. The herbal drink has been found to possess considerable antioxidant capacity as determined by ABTS, DPPH and FRAP assays. The developed herbal drink has to be invariably stored under refrigerated conditions in order to prevent degradation of the phytochemicals analysed as well as to retain its antioxidant capacity.

## Data Availability

The datasets used and/analyzed used in the current study will be made available on reasonable request.

## Abbreviations

ABTS: 2, 2′-azino-bis (3-ethylbenzothiazoline-6-sulfonic acid)
DPPH: 2, 2-diphenyl − 1-picrylhydrazyl
FRAP: Ferric reducing antioxidant power
